# Dynamics of Green Sahara Periods and Their Role in Hominin Evolution

**DOI:** 10.1371/journal.pone.0076514

**Published:** 2013-10-16

**Authors:** Juan C. Larrasoaña, Andrew P. Roberts, Eelco J. Rohling

**Affiliations:** 1 Instituto Geológico y Minero de España, Unidad de Zaragoza, Zaragoza, Spain; 2 Research School of Earth Sciences, The Australian National University, Canberra, Australia; 3 School of Ocean and Earth Science, University of Southampton, National Oceanography Centre, Southampton, United Kingdom; University of Oxford, United Kingdom

## Abstract

Astronomically forced insolation changes have driven monsoon dynamics and recurrent humid episodes in North Africa, resulting in green Sahara Periods (GSPs) with savannah expansion throughout most of the desert. Despite their potential for expanding the area of prime hominin habitats and favouring out-of-Africa dispersals, GSPs have not been incorporated into the narrative of hominin evolution due to poor knowledge of their timing, dynamics and landscape composition at evolutionary timescales. We present a compilation of continental and marine paleoenvironmental records from within and around North Africa, which enables identification of over 230 GSPs within the last 8 million years. By combining the main climatological determinants of woody cover in tropical Africa with paleoenvironmental and paleoclimatic data for representative (Holocene and Eemian) GSPs, we estimate precipitation regimes and habitat distributions during GSPs. Their chronology is consistent with the ages of Saharan archeological and fossil hominin sites. Each GSP took 2–3 kyr to develop, peaked over 4–8 kyr, biogeographically connected the African tropics to African and Eurasian mid latitudes, and ended within 2–3 kyr, which resulted in rapid habitat fragmentation. We argue that the well-dated succession of GSPs presented here may have played an important role in migration and evolution of hominins.

## Introduction

Debate about the role of climate variability in hominin evolution, through its impact on landscapes, has largely neglected North Africa because of the present-day hyper-arid nature of the Sahara and the lack of a fossil record comparable to that of East and South Africa [1], [2]. However, growing evidence indicates that wetland-spotted savannah landscapes spread throughout the Sahara during past periods of enhanced monsoonal precipitation back to the late Miocene [3]–[40], thereby enabling its occupation by hominins [7], [9], [11]–[14], [19], [24], [31], [41]–[50]. These so-called “green Sahara” periods (GSPs) are important for paleoanthropology because most East African hominin sites back to the late Miocene have been related to savannah ecosystems dominated by either open [51] or mosaics of open and closed (often gallery forest) landscapes [52], [53] with permanent freshwater sources [52]–[54]. The Sahara has a massive size compared with the rest of tropical Africa, and its current hyperaridity effectively blocks the gateway to Eurasia. Understanding the timing, extent and duration of GSPs is therefore crucial for a biogeographical approach in studies of hominin evolution to complement a meagre hominin fossil record that represents only a small fraction of their potential biogeographic range [55]. Such recognition has been hindered by: 1) lack of knowledge of the timing, extent and duration of GSPs beyond two of the most recent (Holocene and Eemian) humid periods; and 2) uncertainties about ecological aspects of past Saharan savannah landscapes that are required to assess biome habitability by hominins (except for the Holocene GSP).

Although landscape variability can be established from continental sediments scattered throughout the Sahara, such records provide only a fragmentary view that becomes scarcer and more difficult to date with increasing age. Here we combine continental and marine records of paleoenvironmental variability at different latitudinal bands to provide insights into the timing and duration of GSPs at timescales associated with hominin evolution. By considering the main climatological determinants of woody cover in tropical Africa [56], we translate paleoprecipitation estimates for well-known, representative GSPs (Holocene and Eemian) into specific ecosystems within the savannah biome inhabited by hominins [51]. We combine this information with estimates of hominin dispersal rates [57] and life history data [58] to put biogeographical constraints on hominin population dynamics and evolution in tropical Africa back to 8 million years ago (Ma).

## Materials and Methods

### North African Climate and Vegetation

**Figure 1:**
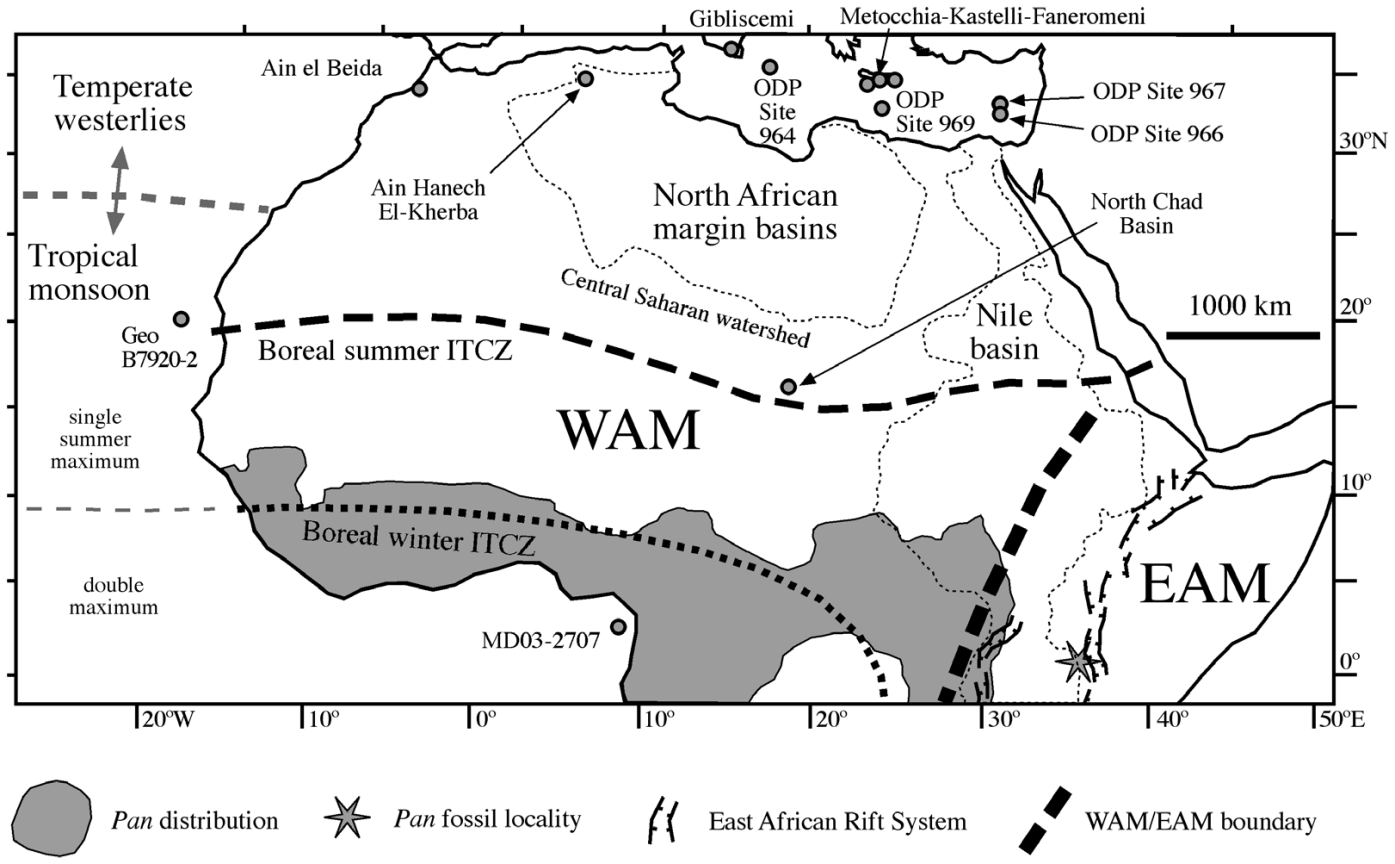
Map of North Africa with relevant meteorological and physiographic features. Meteorological features are after ref. [4], the location of normal faults in the East African Rift System (EARS) is after ref. [88], and the extents of the Nile and North African margin basins are after refs. [8], [78]–[80]. The map includes the present-day distribution (grey shading) and occurrence of fossil remains (star) of chimpanzees (*Pan*) [69], and the locations of records mentioned in the text. WAM: West African monsoon; EAM: East African monsoon; ITCZ: Inter-tropical convergence zone.

**Figure 2:**
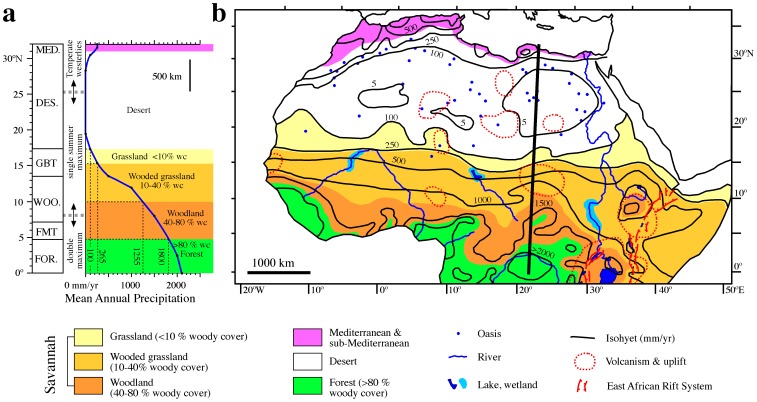
Present day meteorology and vegetation in North Africa. (a) Latitudinal distribution of present-day vegetation belts (MED.: Mediterranean and sub-Mediterranean; DES.: desert; GBT: grassland, bushland and thicket; WOO: woodland; FMT: forest mosaics and transitions; FOR.: rain forest) [60], and mean annual precipitation (MAP) and meteorological elements [3], [4], [59] projected onto a cross-section along the eastern Sahara (dark N-S line in b). (b) Map of the main physiographic and tectonic [88], [97] elements. Coloured vegetation belts are based on a structural classification of vegetation (indicating percentages of woody cover: %wc) using MAP values and main climatological determinants on tropical African biomes [56].

Climatic conditions in North Africa are dictated by the position of the continent around the equator and by regional factors such as topography [4], [59] (Figures 1, 2). North Africa has a narrow fringe with Mediterranean temperate climates in which westerly low-pressure systems (Figure 1) bring mean annual precipitation (MAP) in excess of 250 mm/yr, mainly during boreal winter and spring (Figure 2). Although this precipitation may reach to latitudes of 25°N in extreme events [4], it normally rapidly decreases to less than 100 mm/yr at distances of 100–200 km from the coast.

Tropical Africa has a monsoonal climate in which precipitation is governed by seasonal changes in the differential heating over land and the neighbouring oceans [4], [59] (Figure 1). Equatorial regions receive the most precipitation (>2000 mm/yr) due to their year-round location within the reaches of the boreal summer and winter meteorological equator (the Intertropical Convergence Zone), which drives precipitation with two main wet seasons governed by the West African Monsoon (WAM) (Figures 1, 2). Northward migration of the WAM front into North Africa during boreal summer (Figure 1) drives a pronounced progressive decrease in monsoonal rainfall from ∼1500 mm/yr in near-equatorial regions down to ∼100 mm/yr at around 18°N (Figure 2). Today, WAM rains reach latitudes of up to 25°N during exceptional events [4]. The high topography associated with the East African Rift System (EARS) and a different moisture source, the Indian Ocean, drive the so-called East African Monsoon (EAM) [4], [59] (Figure 1). The broad zonal precipitation pattern observed in Africa north of the equator (Figure 2) is disrupted in East Africa by a rain-shadow effect, which causes reduced precipitation throughout the region, including equatorial areas (500–1500 mm/yr) (Figure 2).

Present-day vegetation in tropical North Africa is influenced by the prevailing climate [60] (Figure 2). Evergreen rainforest occupies the wettest regions around the equator and near the Atlantic coast in West Africa (FOR in Figure 2). The driest part of North Africa (the Sahara Desert) is largely devoid of vegetation with the exception of wadis, oases and mountain areas, where there is sparse vegetation (DES in Figure 2). Between the rainforest and dry desert lies the savannah, which includes three main belts that reflect the gradient of northward-decreasing rainfall. These belts include: 1) a narrow fringe on the periphery of the rainforest that extends from the Atlantic coast to the great lakes region in East Africa and that consists of a mosaic of semi-evergreen forest, woodlands and secondary grasslands (FMT in Figure 2); 2) a grassland, bushland and thicket fringe along the southern margin of the Sahara throughout much of North and East Africa (GBT in Figure 2); and 3) an intermediate woodland belt that lies between the two other ecosystems (WOO in Figure 2). Superimposed on this pattern are the montane forests, bushlands and shrublands that occupy mountain regions of East and North Africa.

### Chronology of Saharan Pleistocene-Holocene Wetter Conditions

We draw on a recent compilation of lacustrine, palustrine and fluvial sediments that are indicative of wetter Holocene conditions throughout the Sahara south of 28°N [6]. The dataset includes 1237 radiocarbon- and luminescence-dated sediments, which have been grouped in three latitudinal bands (13–18°N, 18–23°N and 23–28°N) to examine spatial variations in wetter conditions during the Holocene GSP in response to monsoon dynamics.

For earlier GSPs, we have compiled 136 ages for sediments indicative of wetter conditions throughout the Sahara south of 28°N (Algeria, Chad, Egypt, Libya, Mali, Mauritania and Sudan) [11]–[31]. Of these sediments, 116, 16 and 3 have been dated using uranium-series, luminescence, and electron spin resonance methods, respectively. An additional age derives from ^40^Ar/^39^Ar dating of meteorite impact glass embedded in lacustrine sediments in the Dakhleh paleolake (Egypt) [32]. We have excluded 15 additional ages for which only minimum estimates (of up to >450 kyr) were provided [12], [13], [17], [19], [21], [22]. The main difference between our data set and those recently published by Smith (ref. [9]) and Drake et al. (ref. [10]) is that ours excludes sediments located north of 28°N to isolate signals due to monsoon dynamics.

**Figure 3:**
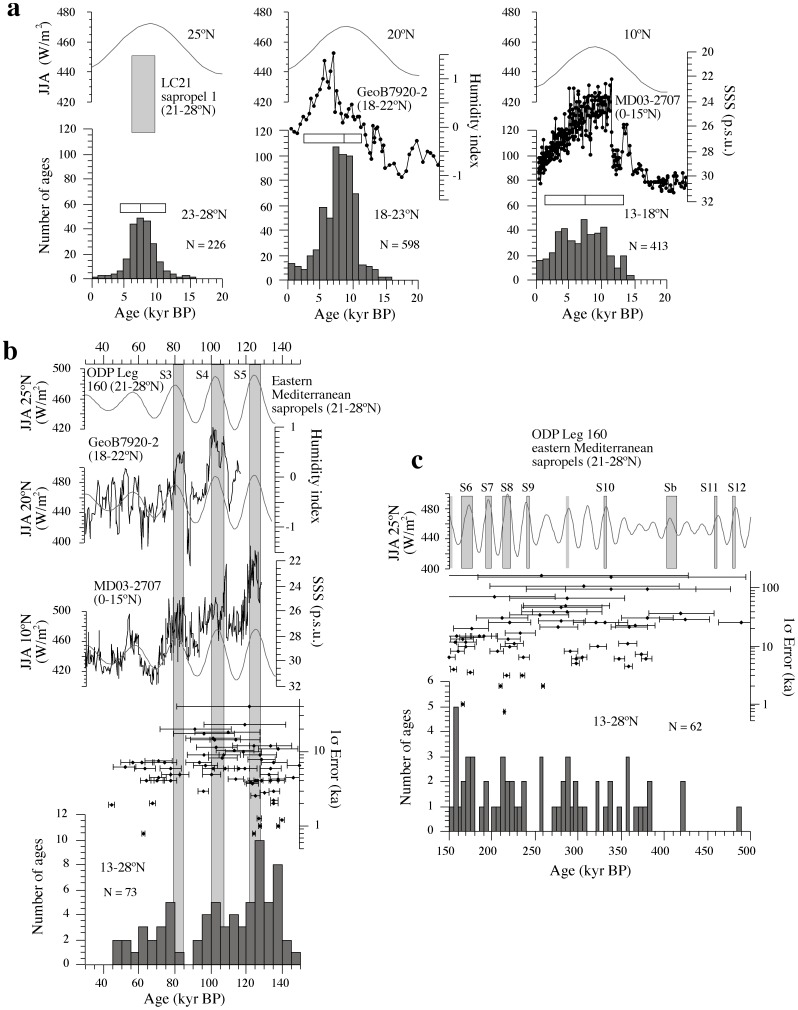
Late Pleistocene-Holocene climate variability in North Africa. (a) Histograms of ages of continental sediments indicative of wetter conditions at different latitudinal bands across North Africa between 0 and 16 kyr BP [6], plotted with marine records that reveal North African climate variability at comparable latitudes (sea surface salinity (SSS) from core MD03-2707 [61], humidity index from core GeoB7920-2 [62] and sapropel record from ODP Leg 160 [63], [64] and summer insolation curves (June, July, August; JJA) at 10°, 20° and 25°N [107]. Bars indicate the 5 and 95 percentiles around the median of the histograms. (b) Histogram of ages of continental sediments indicative of wetter conditions throughout the Sahara between 30 and 150 kyr BP, with SSS from core MD03-2707 [61], humidity index from core GeoB7920-2 [62] and the sapropel record from core ODP Leg 160 [63] and their relevant summer insolation curves [107]. The chronology of sapropels is after ref. [65]. (c) Histogram of ages of continental sediments indicative of wetter conditions throughout the Sahara between 150 and 500 kyr BP, plotted with the eastern Mediterranean sapropel record from ODP Leg 160 [63] and summer insolation at 25°N [107]. Unlabelled sapropels correspond to those erased by post-depositional oxidation [63].

We combine the terrestrial data with three marine middle Pleistocene to Holocene climate records that are widely recognized as indicative of paleoclimate conditions at different latitudinal bands (Figures 1, 3): 1) the sea surface salinity (SSS) record of core MD03-2707, which responds to riverine runoff in the Niger and Sanaga river catchments, and integrates WAM signals between 5° and 15°N [61]; 2) the humidity index from core GeoB7920-2, which responds to changes in eolian and fluvial contributions from the Sahel/Sahara boundary region, and integrates WAM signals between 18° and 22°N [62]; and 3) the sapropel record of sites 964, 966, 967 and 969 of the Ocean Drilling Program Leg 160, which is representative of sapropel deposition throughout the eastern Mediterranean Sea [63]. The later responds to a combination of enhanced Nile and other riverine discharge along the wider African-Mediterranean margin, which integrates WAM signals north of the central Saharan watershed at ∼21°N [63]–[66]. We select these records because they provide specific information about monsoonal precipitation at different locations around North Africa that can be directly compared with their terrestrial counterparts.

### Biogeography of Hominins in Tropical Africa

Recently, hominin habitats have been characterized by translating δ^13^C data from paleosol carbonates into a structural definition (in terms of woody cover) of the different ecosystems with which fossil hominins are associated [51] (Table 1). Accordingly, nearly all (>99%) East African hominins are associated with three ecosystems within the savannah biome [51], which is defined as a mixed tree-grass system characterized by a discontinuous tree canopy (up to 80% of woody cover) in a continuous C_4_ grass layer [67]. Soil carbonates mainly form when evaporation exceeds precipitation, therefore this method may provide a view of the amount of tree cover associated with hominin sites that is somewhat biased toward drier habitats [68]. However, qualitative multiproxy paleoenvironmental reconstructions imply a recurrent association of hominin sites with open landscapes or with landscape mosaics including grasslands, woodlands and forests typically associated with permanent freshwater lakes, rivers and springs [52]–[54]. Regardless of whether this association is genuine or the result of temporal or spatial averaging due to taphonomic processes [52], [53], it clearly illustrates a link between hominin sites and dynamic savannah landscapes with variable woody cover that includes riparian forests along freshwater sources. This independent support for the quantitative reconstructions of Cerling et al. (ref. [51]) enables identification of hominin habitats back to at least 6.5 Ma despite variable background temperature and atmospheric CO_2_ conditions, which also affect vegetation.

**Table 1:** Reconstructed woody cover and estimates of hominin sites associated with different tropical ecosystems (after ref. [51]), along with our calculated mean annual precipitation (MAP) values for such ecosystems.

Biome	Ecosystem	Woody cover (%)	Hominin sites (%)	Inferred MAP (mm/yr)
	Grassland	<10	10	100–265
Savannah	Wooded grassland	10–40	60	265–1255
	Woodland	40–80	29	1255–1800
Tropical forest	Forest	>80	<1	>1800

Despite the well-established link between vegetation and precipitation in tropical Africa [60] (Figure 2), the main climatological determinants of woody cover in tropical Africa have only recently become established, based on a comparison of daily precipitation and woody cover records derived from satellite data [56]. A least-squares regression between F_c_ (fraction of woody cover), P_w_ (mean wet season precipitation), and α_w_ (average wet season storm intensity, defined by normalizing P_w_ to the duration of the wet season) provides a robust statistical relationship (r^2^ = 0.65) between F_c_ and the quantity and intensity of rainfall according to [56]:
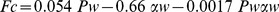



Across tropical Africa, around 79% of the annual rainfall arrives in the wet season, which has an average duration of 172 days [56]. Considering these average estimates for P_w_ and α_w_, this equation can be used to estimate the percentage of woody cover from MAP data. We have inverted the equation to calculate the MAP value at which the fraction of woody cover attains certain values. By setting the fractions of woody cover to 10, 40 and 80%, we can estimate the isohyets that delineate the different structurally-defined savannah ecosystems with which nearly all East African fossil hominin sites are associated [51] (Table 1). Based on the present-day coincidence of the northern boundary of grasslands with the 100 mm/yr isohyet, and given that this boundary is not defined in terms of woody cover, this MAP value has been used to delineate the boundary between savannah and desert. Since the equation does not capture the full range of rainfall variability for the highest fractions of woody cover [56], we set the limit between savannah and rainforest at the northernmost rainforest boundary at ∼1800 mm/yr of precipitation. Good coherency between structural types of savannahs determined from MAP values and the vegetation types of White (ref. [60]) (left and middle panels in Figure 2) validates our approach.

Next, we consider the combined distribution of different savannah habitats throughout tropical Africa as the biogeographic range of hominins. The need for such an approach is illustrated by the distribution of extant and fossil chimpanzees and gorillas. While the only secure report of fossil chimpanzee remains to date comes from middle Pleistocene successions at Tugen Hills (Kenya) in the EARS, the present-day (before historical habitat disruption) distribution of chimpanzees spans a broad belt across equatorial Africa (between 10°S and 15°N) that includes rainforest, woodland and dry savannah, but that does not extend east into the EARS [69] (Figure 1). The case for gorillas is even more marked, with no secure identification of fossil remains so far. Failing to recognise the biogeography of chimpanzees and gorillas with respect to their relevant biomes, and relying only on their fossil remains, would inevitably lead to erroneous inferences concerning their past distribution, habitats and population dynamics. The same holds for hominins, so that ignoring past habitats suitable for their occupation would result in an incomplete environmental backdrop to frame their evolution. We attribute the scant occurrence of fossil hominins in North Africa as due to geological conditions unfavourable for creating sediment accommodation space (as opposed to the EARS and some South African karst systems [2]) and to sampling bias (historical focus on East and South Africa [55]).

### Distribution of Hominin Habitats during Representative GSPs

**Figure 4:**
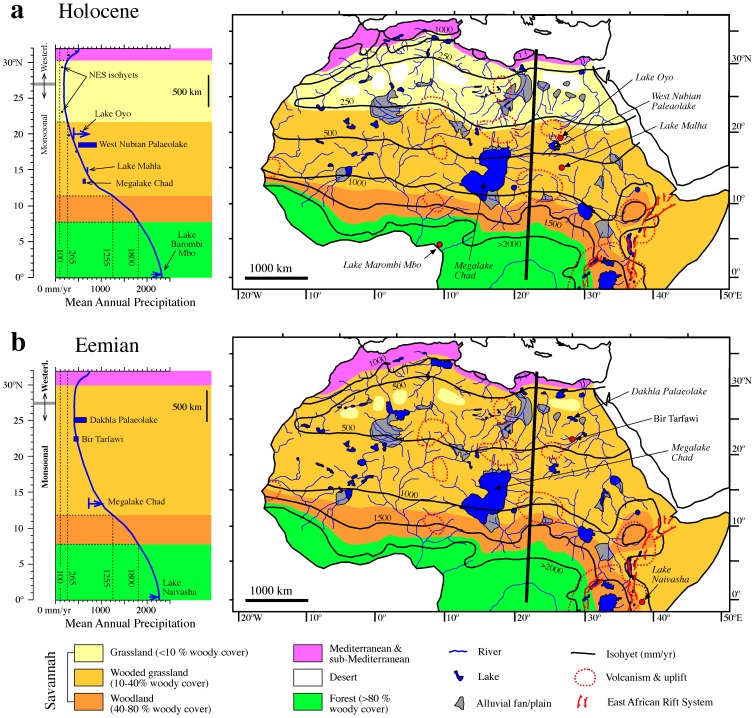
Reconstruction of North African vegetation during past green Sahara periods. (a) Estimated and reconstructed MAP for the Holocene GSP (6–10 kyr BP) projected onto a cross-section along the eastern Sahara (left panel) and map view of reconstructed MAP, vegetation and physiographic elements [7], [8], [11], [45] (right panel). (b) Estimated and reconstructed MAP for the Eemian GSP (122–128 kyr BP) projected onto a cross-section along the eastern Sahara (left panel) and map view of reconstructed MAP, vegetation and physiographic elements [14], [15], [44], [45] (right panel).

Paleoprecipitation estimates for the Holocene GSP (∼11 to 6 ka) based on multiple geological, archeozoological and archeobotanical data indicate a MAP of around 100 mm/yr at the core of the northeastern Sahara (between 23° and 29°N) and of 450 mm/yr at around 18°N [41]. Further south, pollen data from lakes Oyo (∼19°N) and Malha (∼15°N) suggest MAP values of ≥400 mm/yr [33] and 700 mm/yr [34], respectively. Paleohydrological modelling of the West Nubian (18°–19°N) and Chad (10°–18°N, centered at 14°N) megalakes points to MAP values of 500–900 mm/yr [35] and 600–650 mm/yr [36], respectively. An increase in MAP of 250 mm/yr is indicated in present-day rainforest areas of equatorial West Africa (∼5°N) on the basis of pollen data from Lake Barombi Mbo [70]. These estimates have been projected into a N-S section across the eastern Sahara and into equatorial Africa (Figure 4a). We then fitted a profile of these estimates along the same cross-section used for present-day precipitation variations in Figure 2. From this new profile we then established the positions of the 250, 500, 1000, 1500 and >2000 mm/yr isohyets (Figure 4a). Given the lack of error estimates in most paleoprecipitation reconstructions, we have drawn paleoprecipitation throughout North Africa based on a broad zonal pattern with a curvature in East Africa and taking into account the influence of major topographic features. This approach also affects the 265, 1255 and 1800 mm/yr isohyets that delineate the grassland/wooded grassland, wooded grassland/woodland, and woodland/forest boundaries, respectively.

Paleoprecipitation estimates for the Eemian are available from paleohydrological modelling of the Dakhla (∼26°N) and Chad megalakes and from fossil vertebrate assemblages from Bir Tarfawi (22°N) (Figure 4b). Paleohydrological modelling of the Dakhla and Chad megalakes suggests MAP values of 410–670 mm/yr [16] and ≥730 mm/yr [39], respectively. Fossil faunas from Bir Tarfawi indicate a MAP value of ≥500 mm/yr [40]. We are unaware of Eemian paleoprecipitation reconstructions from present-day equatorial West African rainforest areas. We have, therefore, considered a MAP increase of 270 mm/yr reported from paleohydrological modelling of Lake Naivasha [71] (Figure 4b) to be representative of Eemian precipitation changes in equatorial Africa. We then followed the same procedure used for the Holocene to reconstruct MAPs and ecosystem distribution throughout North Africa during the Eemian GSP.

## Results and Discussion

### Middle Pleistocene to Holocene Climate Variability in North Africa

In the southern Sahara (13–18°N), ages of latest Pleistocene to Holocene sediments indicative of humid conditions cluster around 7.5 ka, with a wide distribution (90% of ages are between 1.5 and 13.5 ka) that matches the broad interval of increased monsoonal precipitation between 0° and 15°N [61] and encompasses the local summer insolation peak (Figure 3a). In the central Sahara (18–23°N), the distribution of ages is narrower around 8.5 ka (90% of ages are between 2.5 and 11.5 ka), in correspondence with a shorter period of insolation-driven increased monsoonal precipitation at around 20°N [62]. Further north (23–28°N), ages cluster more tightly around 7.5 ka (90% of ages are between 4.5 and 11.5 ka) and broadly coincide with deposition of an organic-rich layer in the eastern Mediterranean Sea known as sapropel S1, and which reflects monsoon runoff flooding from North Africa into the eastern Mediterranean Sea [64], [65]. These data indicate a progressive (2–3 kyr) northward expansion and southward retraction of monsoonal precipitation at the onset and termination of the GSP, respectively. Monsoon-driven wetter conditions throughout the Sahara peaked over a ∼4 kyr period that is broadly marked by deposition of sapropel S1. Both the mid-point of sapropel S1 and clusters of sediments indicative of wet conditions in the Sahara lag by 1.5–3 kyr relative to the insolation peak, which has been attributed to groundwater recharge preceding development of large lakes at the onset of the GSP [6] and arrival of runoff to the Mediterranean Sea.

Between 30 and 150 ka, scarcity of dated terrestrial sediments prevents separation of ages within different latitudinal bands. Due to potential prolonged lake and spring activity related to groundwater discharge and to the errors (typically >5 kyr) associated with most published ages, ages of sediments indicative of wetter conditions are scattered (Figure 3b) [9], [10]. Field evidence indicates that these sediments are grouped into genetically related units that are typically separated by eolian deflational surfaces [8], [11], [12], [13], [15], [17], [24]. This points to prevailing hyperarid conditions punctuated by short wetter spells. With these caveats in mind, we identify three prominent clusters of ages at around 80, 105 and 125 that we interpret as genuine periods of widespread wetter conditions in the Sahara in agreement with other studies [9], [10]. These three clusters coincide, when considering chronological errors, with distinctive peaks in the SSS record of core MD03-2707 [61], the humidity index of core GeoB7920-2 [62], sapropels S3 to S5 in the eastern Mediterranean [63], [65], and local summer insolation maxima (Figure 3b). These data corroborate the causal link between monsoon dynamics and green Sahara conditions, which peaked throughout the Sahara during deposition of eastern Mediterranean sapropels. The decreasing duration of wet periods from south to north, as portrayed by the marine records (Figure 3b), further highlights the time-transgressive onset and demise of GSPs. Smaller clusters of ages between 40 and 60 ka broadly coincide with wetter conditions in core MD03-2707 [61] and other records that indicate enhanced precipitation at Africa around 10°N [72]. These ages also coincide with slightly wetter conditions in core GeoB7920-2 [62], but not with any eastern Mediterranean sapropel (Figure 3b). This demonstrates the occurrence of other periods during which monsoonal precipitation was enhanced, but which lacked the power to drive expansion of savannah landscapes beyond the central Sahara. The low amplitude of insolation peaks at around this time (Figure 3b) suggests a link with cold sea surface temperatures in the North Atlantic, which exerted a dominant role on North African humidity changes at times of weaker monsoon activity [10], [47], [61], [72]. The interval between 150 and 500 ka is marked by fewer continental sediment ages, which become progressively scarcer back in time (Figure 3c) due to protracted erosion of older sediments [9], [10]. The errors associated with the ages of these sediments are larger (>10 kyr), which prevents correlation of clusters of ages to deposition of eastern Mediterranean sapropels (Figure 2c). Despite this, ages cluster with a ∼20 kyr frequency that is indicative of control by insolation-driven enhanced monsoon rainfall [9], [12], [23]. Based on the lesson learned from the much better documented late Pleistocene-Holocene period, we interpret that short-lived middle Pleistocene wet periods in the Sahara were also concurrent with eastern Mediterranean sapropel deposition during GSPs.

### Timing of green Sahara periods since 8 Ma

Our compilation of middle Pleistocene to Holocene paleoenvironmental records (Figure 3) indicates that marine records provide excellent documentation of circum-Saharan monsoon variability, which complements a continental record that becomes more scarce, fragmentary and difficult to date at older times. Prior to 130 ka, the sapropel record provides the only continuous and precisely (astronomically) dated archive with the potential for identifying peak savannah expansions at timescales associated with hominin evolution.

Sapropel formation has been traditionally attributed to increased Nile runoff during summer insolation maxima [73]. The main sources for Nile runoff are the highlands of Uganda and Ethiopia in East Africa (Figure 1); some authors attribute increased Nile runoff periods to rainfall driven mainly by the WAM [73], while others relate them to EAM dynamics [74]. However, δ^18^O data from sapropels [75], [76] demonstrate that the lightest values are typically found off Libya rather than off the Nile mouth. Significant northward penetration of the WAM summer front beyond the central Saharan watershed (∼21°N) (Figure 1) must have occurred simultaneously with enhanced Nile runoff to allow direct drainage of low-δ^18^O monsoonal precipitation into the eastern Mediterranean [77] (Figure 1). Such massive drainage was channelled toward the gulfs of Sirte and Gabes through now-extinct river systems identified along the North African margin [8], [78]–[80] (Figure 1), as demonstrated with neodymium isotope ratios [44]. Penetration of the summer WAM front as far as ∼25°N during GSPs, which would have enabled substantially enhanced monsoon runoff into the eastern Mediterranean, is evidenced by widespread formation of lake and fluvial systems between 20° and 28°N that are disconnected from more southerly (monsoonal) and northerly (Mediterranean) hydrological systems [4], [6], [8]–[12], [14]–[20], [44], [45], [78]–[80]. This conclusion is further supported by modelling results that highlight the positive feedbacks between lake formation and northward expansion of monsoon rainfall [38]. Enhanced WAM (as opposed to EAM) precipitation during GSPs also provides an explanation for enhanced pedogenic activity [81] and dampening of dust production [66] throughout the Sahara during periods of sapropel formation. Such a prominent role of the WAM as the main driver of GSPs explains the accumulation of sapropels back to 10 Ma, under the same scenario of enhanced monsoonal discharge during local summer insolation maxima [63], [82], [83], well before the earliest clear evidence for a river connecting the East African tropical regions with the Mediterranean Sea across Egypt sometime between the latest Miocene (ca 6 Ma [79]) and the early Pliocene (4–5 Ma [84]).

**Figure 5:**
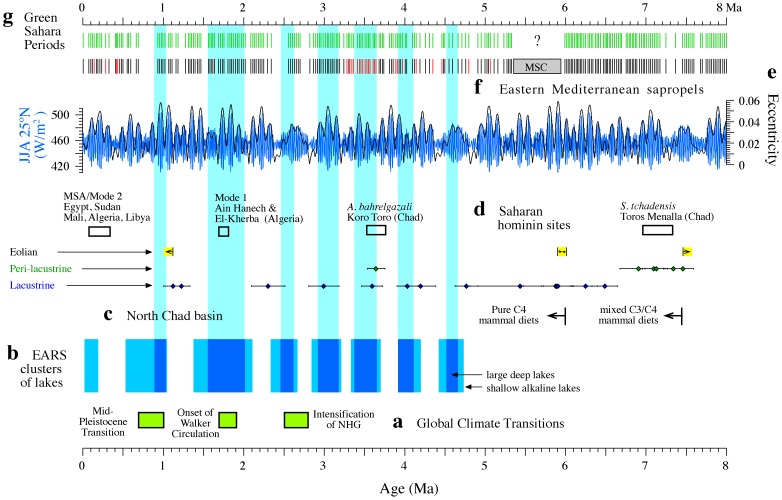
Saharan climate and hominin occupation 8 Ma to present. (a) Chronology of global climate transitions [88] (NHG: northern hemisphere glaciation). (b) Chronology of East Africa Rift System (EARS) lake periods [88]. Formation of large lakes was prevented by the lack of full-graben basin morphologies before 5 Ma. Vertical shaded bars indicate periods with large, deep, freshwater lakes. (c) Chronology of lacustrine, peri-lacustrine and eolian sediments in the North Chad Basin [48] plotted with earliest evidence for mixed C_3_/C_4_ and pure C_4_ mammal diets in the basin [96]. (d) Chronology of Saharan hominin occupation sites [11], [12], [14], [47], [48], [50]. (e) Variations in summer insolation (June, July, August; JJA) at 25°N and eccentricity back to 8 Ma [107]. (f) Composite eastern Mediterranean sapropel record from ODP Leg 160 sites [63] and land sections at Gibliscemi, Metochia, Kastelli and Faneromeni [85], [86] (red bars indicate oxidized sapropels). (g) Green Sahara periods back to 8 Ma inferred from the eastern Mediterranean sapropel record.

Based on the mechanistic link established here between sapropels and GSPs, we take eastern Mediterranean sapropels [63], [85], [86] as markers of over 230 GSPs back to 8 Ma (Figure 5f,g). *In situ* evidence for recurrent GSPs over millions of years is provided, despite its fragmentary nature, by the lacustrine, peri-lacustrine and eolian sedimentary record of the North Chad Basin, which demonstrates an alternation of humid and dry conditions since ∼8 Ma [48] (Figure 5c). The only gap in sapropel sedimentation is observed between ca 5.3 and 6 Ma, due to disruption of normal marine sedimentation during the Messinian Salinity Crisis (MSC) (Figure 5f). Given the link between GSPs and insolation maxima, we infer that at least the most prominent local summer insolation maxima also led to GSPs between 5.3 and 6.6 Ma. This inference is supported by observation of an undisturbed pattern of North African climate variability across the MSC [87].

Overall, we infer that WAM variability led to recurrent GSPs back to 8 Ma (Figure 5g), with GSPs forming 400-kyr and 100-kyr clusters that attest to the impact of eccentricity modulation of precession (and hence of insolation) on monsoon variability [63], [82], [83], [85], [86] (Figure 5a,e,f). A less regular sapropel pattern after 0.9 Ma [63], [66] suggests that GSPs were somewhat less frequent after development of large polar ice caps during the Mid-Pleistocene Transition (Figure 5a,f,g). The notion of eccentricity modulation of precession as the main driver of WAM variability provides strong support for a similar influence on the EAM, given that phases of expanded lakes in the EARS coincided with 400 kyr eccentricity maxima [2], [88], [89] (Figure 5b) during which moisture availability was driven by insolation changes at precessional timescales [2], [90]. Simultaneous response of WAM and EAM systems to insolation forcing has been demonstrated based on the synchronicity of periods of higher water availability in East Africa and of eastern Mediterranean sapropel deposition at least at 0.1–0.2 Ma [91] and 1.85–1.95 Ma [92].

### Landscape Composition and Hominin Occupation during Green Sahara Periods

We translate Holocene precipitation estimates [33]–[36], [41], [70] into percentages of woody cover in tropical savannahs to infer the presence of wooded grasslands (10–40% woody cover) between 10° and 20°N and grasslands (woody cover <10%) north of ∼22°N (Figure 4a). Although more paleoprecipitation estimates are needed to provide a detailed picture of the distribution of different habitats, the proposed distribution is consistent with both paleoenvironmental evidence across North Africa [4], [5], [37], [41]–[43] and climate modelling data [38]. Enhanced precipitation led to development of interconnected rivers, lakes, playas and terminal alluvial fans throughout the Sahara [3]–[7], [11], [35], [41]–[43], [45], [80] (Figure 4a). This drainage network enabled expansion of Sudanian and Sahelian vegetation into the desert as gallery forests [5] that coexisted with grasslands [42] to create a biodiverse ecosystem with no present analogue [5]. Paleontological and archeological data indicate widespread trans-Saharan dispersals of aquatic and savannah faunas along interconnected drainage systems and grasslands [45]. Field [41] and modelling data [38] suggest precipitation <100 mm/yr in some areas north of ∼28°N, especially in the northeast Sahara. This points to continued desert conditions in isolated regions, separated by major rivers and lake systems. Paleoclimatic and modelling data also indicate intensification and southward displacement of mid-latitude cyclones down to ∼30°N during GSPs [9], [10], [81], [93], which brought increased rainfall to the Mediterranean rainfall belt of the North African margin [38], [41]. This must have resulted in slight southward expansion of Mediterranean vegetation and in the narrowing of isolated arid regions between ∼28° and ∼30°N (Figure 4a).

The scarcer paleoprecipitation estimates available for the Eemian GSP (122–128 kyr BP) [16], [39], [40], [71] point to markedly increased precipitation across North Africa compared to the Holocene GSP (Figure 4a, b), as is evident also from quantifications of monsoonal runoff into the eastern Mediterranean [44], [77] and from modelling data [39]. Eemian GSP paleoprecipitation points to wooded grassland occurrences (10–40% woody cover) across the Sahara, which is supported by the occurrence of tropical humid plants (*Ficus*, *Celtis*, and ferns) and subtropical vertebrate fauna as far north as 23–26°N in the Western Desert [14], [40]. Given these overall wetter conditions, it is likely that the discontinuous arid belt between the wooded grasslands to the south and Mediterranean vegetation to the north was narrower and less arid compared to the Holocene. Although paleohydrological information on the Eemian GSP is not as abundant as for the Holocene GSP, evidence for megalakes and fluvial activity throughout the Sahara is compelling [9]–[16], [44]–[46], [80] so that reconstruction guided by the Holocene GSP is tenable [45] (Figure 4b). Field data indicate a marked size increase of older Saharan paleolakes, which attest to a significant progressive precipitation decrease during peak GSPs since 500 ka [8], [9], [12], [15]. This is supported by pollen records off northwest Africa [94], and indicates that the Eemian GSP can be taken as representative of other middle Pleistocene GSPs.

Development of fluvial networks and megalakes throughout the Sahara during Messinian, Pliocene and lower Pleistocene times [8], [78], [80] provides support for recurrent GSPs since the late Miocene. Paleoprecipitation estimates are not available for such older GSPs, but their paleolakes were considerably larger than for middle Pleistocene to Holocene GSPs [8]. Although the complex interplay of drainage networks with volcanic, tectonic and geomorphic features may have influenced lake development and size [8], the extension of older large paleolakes to as far north as 28°N suggests that conditions were wetter during older peak GSPs [8], [80]. This is consistent with the more regular sapropel pattern before 0.9 Ma [63], [66], which suggests that WAM penetration over the Sahara was favoured before development of large polar ice caps around the Mid-Pleistocene Transition. Regardless, the continued occurrence of desert plant pollen in marine sediments off northwest Africa since at least the middle Pleistocene [94], coupled with the persistence of arid adapted mammals in the Sahara back to ∼5 Ma [95], suggests that the discontinuous arid belt between the subtropical savannah and the Mediterranean eco-region never disappeared completely. It therefore appears that rivers emanating from the central Saharan watershed must have played a key role in enabling dispersals across the Sahara during GSPs, especially during relatively drier GSPs such as the Holocene.

Widespread archeological evidence indicates human occupation of the entire Sahara during the Holocene GSP [7], [11], [41]–[43], [45]. Earlier and Middle Stone Age remains are far less abundant than Neolithic ones, but their association with fluvial, lacustrine, and spring deposits compellingly links human occupation of the Sahara with middle and late Pleistocene GSPs [9], [12]–[14], [46], [47], especially during the Eemian [9], [11]–[14], [44], [45], [47]. Although archeological remains scattered throughout the Sahara provide evidence of human occupation before 0.5 Ma [49], well dated sites are restricted to the Earlier Stone Age archeological remains in Ain Hanech and El-Kherba in Algeria (1.7–1.8 Ma) [50] and to fossil hominin remains in Koro Toro and Toros Menalla (North Chad Basin), which date to 3.5–3.7 Ma and 6.9–7.3 Ma, respectively [48] (Figure 5d). All of these sites have been linked either to open landscapes that included permanent freshwater lakes and streams (Ain Hanech and El-Kherba) [50], or to mosaics of open to gallery forest landscapes with nearby permanent lakes that evolved from and to desert conditions (Koro Toro and Toros Menalla) [53], [48]. Paleoenvironmental reconstructions of these sites indicate GSP-like conditions that were much wetter than present-day arid climates. Evidence from these sites therefore strongly support a case for recurrent hominin occupation of the Sahara during GSPs since the late Miocene. Within chronological uncertainties, these hominin occupations during GSPs coincided with large East African lake phases and 400-kyr eccentricity maxima (Figure 5).

Mammal teeth carbonate δ^13^C data from the North Chad Basin demonstrate the occurrence of C_4_ grasses by 7.45 Ma [96], which implies the episodic presence of true savannah [67] in the Sahara since at least that time. Pollen data indicate uniquely biodiverse savannah landscapes [5] during GSPs. Saharan grasslands and wooded grasslands with gallery forests along interconnected drainage systems during GSPs back to 8 Ma therefore provide an ecological context to hominin occupations that is comparable to the landscapes associated with most East African hominin sites back to the late Miocene [50]–[53]. Central areas of the Sahara were also affected by local tectonic uplift and volcanism during the late Neogene and Quaternary [97], which would have contributed to creation of dynamic landscapes favourable for hominin occupation as they do in East and South Africa [97]. From a biogeographic perspective, supported by periods with incontestable evidence for hominin presence in the Sahara [7], [9], [11]–[14], [41]–[50], it appears that the multiple GSPs since 8 Ma represented massive episodic expansions of prime hominin habitats into North Africa.

### Implications for Hominin Evolution

Our compilation of marine and continental paleoenvironmental data (Figure 3) indicates a time-trangressive onset of GSPs from south to north over periods of a few thousand years. Estimated dispersal rates of hominins and other large mammals are rapid, of the order of 13–21.5 km/yr [57]. Even if Saharan reoccupation by hominins and large mammals during GSPs was limited by the lower dispersal rate of tropical plants (0.5 km/yr [5]), then colonization throughout North Africa would still have been achieved within 2–3 millennia of initial savannah expansion. Values of ∼15 years for the age at first birth [58] assumed for hominins, along with observed durations of peak green Sahara conditions of 4–8 millennia (based on typical sapropel durations [64], [65], [82], [98]), suggest that hundreds of generations of hominins could have thrived in North Africa during each GSP. Subsequent GSP terminations would have caused major population collapses and displacements, and habitat fragmentation with associated potential for isolating small residual populations. These conditions would be conducive to genetic drift and strong selection pressures, possibly to the point of speciation or extinction.

The fossil record attests to frequent connections between East and Northwest African mammal faunas since the late Miocene, with the faunas of the two regions reaching maximum affinity in the middle Pleistocene [99]. Archeological and fossil records also indicate recurrent hominin occupation of the Sahara since the late Miocene [7], [9], [11]–[14], [41]–[50] (Figure 5d). Our corroboration of astronomically-controlled monsoon variability as the main driver of changes in tropical and subtropical African vegetation [2], [47], [88], [89] points to recurrent GSPs as the underlying mechanism that enabled frequent connection between East and Northwest African mammal faunas and recurrent hominin occupation of the Sahara. The fossil record also suggests frequent bidirectional exchanges of large mammal faunas between Africa and Asia since the late Miocene, including hominins [57], [100]. Furthermore, genetic evidence points to recurrent gene flow between African and Asian hominins (modulated by isolation due to distance) after their first out-of-Africa dispersal [101]. In the absence of land bridges across the southern Red Sea after the Late Miocene [102] and of unequivocal evidence for navigation skills before *H. sapiens* [103], GSPs appear to provide (in addition to seafaring along the Red Sea coast) a critical control mechanism for hominin migrations between Africa and Asia through the Levantine corridor after enabling their first out-of-Africa dispersal. This control may be ancient. Recent re-evaluation of the initial out-of-Africa dispersal of the genus *Homo* and its subsequent colonization of Asia, thought to date to around 1.85 Ma [50], [57], suggests that this dispersal may be considerably older, probably between 1.9 and 2.4 Ma [55], [104]. It has been also suggested that *Homo* may have speciated in Asia from an earlier African immigrant hominin, before eventually dispersing throughout Eurasia and back again into Africa [104]. The frequent GSPs that we document prior to 1.85 Ma would have facilitated such earlier, bidirectional dispersals [104] that we tentatively link with GSPs around the eccentricity maxima at 2.1–2.3 Ma (Figure 5e,g). Our interpretation of older GSPs supports the idea that biological evolution or social organization factors, rather than climate [57], may have been limiting to earlier first out-of-Africa dispersal of hominins.

The last four GSPs (at 6–10, 77–81, 102–108 and 122–128 kyr BP) coincided with wetter conditions across North [61], [62], [63], [65] and East Africa [91], the Arabian Peninsula [105] and the Levant [106], due to similar regional climatic responses to enhanced insolation maxima. Over longer timescales, 400-kyr eccentricity changes appear to have caused clusters of GSPs that coincided with large East African lake phases (Figure 5). GSPs, therefore, facilitated critical biogeographic connections within a widespread region with enhanced water availability that stretched from the African tropics to the Eurasian mid latitudes. In addition, North African savannahs during GSPs were larger (∼14×10^6^ km^2^) than East and South African savannahs combined (∼6**×**10^6^ km^2^), so that GSPs represented at least an episodic threefold extension of prime hominin habitats, followed by rapid habitat fragmentation. Overall, Saharan savannahs likely enabled modulation of hominin population dynamics in tropical Africa and controlled accessibility of the Levantine gateway to Asia. Thus, we infer that the recurrent GSPs that we document over the past 8 Myr may have had a large, but underappreciated, potential for driving hominin speciation, extinction, adaptation, and migration.
